# The Effects of Dehydration on Metabolic and Neuromuscular Functionality during Cycling

**DOI:** 10.3390/ijerph17041161

**Published:** 2020-02-12

**Authors:** Francesco Campa, Alessandro Piras, Milena Raffi, Aurelio Trofè, Monica Perazzolo, Gabriele Mascherini, Stefania Toselli

**Affiliations:** 1 Department for Life Quality Studies, University of Bologna, 47921 Rimini, Italy; aurelio.trofe2@unibo.it; 2 Department of Biomedical and Neuromotor Sciences, University of Bologna, 40126 Bologna, Italy; alessandro.piras3@unibo.it (A.P.); milena.raffi@unibo.it (M.R.); monica.perazzolo2@unibo.it (M.P.); stefania.toselli@unibo.it (S.T.); 3 Department of Experimental and Clinical Medicine, University of Florence, AOUC, 50139 Careggi, Florence, Italy; gabriele.mascherini@unifi.it

**Keywords:** bioimpedance, BIVA, body composition, hydration status, phase angle, vector length

## Abstract

This study aimed to determine the effects of dehydration on metabolic and neuromuscular functionality performance during a cycling exercise. Ten male subjects (age 23.4 ± 2.7 years; body weight 74.6 ± 10.4 kg; height 177.3 ± 4.6 cm) cycled at 65% VO_2max_ for 60 min followed by a time-to-trial (TT) at 95% VO_2max_, in two different conditions: dehydration (DEH) and hydration (HYD). The bioelectrical impedance vector analysis (BIVA) and body weight measurements were performed to assess body fluid changes. Heart rate (HR), energy cost, minute ventilation, oxygen uptake, and metabolic power were evaluated during the experiments. In addition, neuromuscular activity of the vastus medialis and biceps femoris muscles were assessed by surface electromyography. After exercise induced dehydration, the bioimpedance vector significantly lengthens along the major axis of the BIVA graph, in conformity with the body weight change (−2%), that indicates a fluid loss. Metabolic and neuromuscular parameters significantly increased during TT at 95% VO_2max_ with respect to constant workload at 65% of VO_2max_. Dehydration during a one-hour cycling test and subsequent TT caused a significant increase in HR, while neuromuscular function showed a lower muscle activation in dehydration conditions on both constant workload and on TT. Furthermore, a significant difference between HYD and DEH for TT duration was found.

## 1. Introduction

Analyzing and monitoring body composition combined with the search for optimal physical condition and recovery of physiological parameters in high-level athletes have always been topics of study for researchers, trainers, and coaches [1,2,3]. For decades, the relationship between hydration status and performance has been closely evaluated, with hydration status being directly linked with physical performance. Many studies have reported the consequences of dehydration on physical and mental levels, highlighting humoral changes and cognitive deficits, which not only compromise normal daily activities but can negatively affect sports performance [4,5,6]. More specifically, it has been shown that the inadequate restoration of fluids during exercise compromises neuromuscular function, increases fatigue perception, reduces technical skills, affecting metabolic and autonomic nervous system parameters [7,8,9,10].

The National Athletic Trainers’ Association (NATA) recommends using a combination of methods to assess hydration status, including body mass change, urine color or urine specific gravity (USG) after first morning void, as well as thirst level to track hydration status [11]. Studies show that only a loss of 1% to 2% of body mass from sweating is enough to compromise physiological functioning and sport performance during exercise. On the contrary, maintenance of body water during exercise is thought to provide protection from thermal injury, reduce physiological strain and maintain or even improve sport performance [11,12]. Recently, it has been shown that the BIVA method is capable of measuring changes in body composition in the short and long term, even after exercise [13,14,15,16]. This method plots the impedance parameters [resistance (R) and reactance (Xc)] standardized for the subject’s height on a graph as a single vector [17], where the shortening or lengthening of vectors represents fluid loss or gain respectively [18,19]. R arises from Extracellular Water (ECW) and Intracellular Water (ICW). Conversely, Xc arises from cell membranes and represents the cell membrane’s quality of taking an electric load and liberate it in a second moment, after a brief delay; it could be compared to a vessel-capacitance-like property [20].

There is still an incomplete picture regarding the loss of fluids due to exercise and its relationship with physical performance variables. Despite the importance of maintaining a euhydrated state, studies have shown individuals are not adequately replacing fluid during exercise [21]. Although many studies have investigated the physiological responses to dehydration [4,5,6,7,8,9,10,11,12], to our knowledge, no research has evaluated the impact of dehydration on both metabolic and neuromuscular variables during performance in active males. Therefore, the purpose of our study was to investigate the effects of progressive dehydration on heart rate (HR), oxygen uptake (VO_2_), energetic cost, and neuromuscular functionality during both submaximal cycling exercise at a constant work rate and subsequent time-to-trial (TT) performance.

## 2. Materials and Methods

### 2.1. Participants

We recruited ten active male subjects (age 23.4 ± 2.7 years; body weight 74.6 ± 10.4 kg; height 177.3 ± 4.6 cm) who volunteered to participate in this study. The following inclusion criteria were used: (1) a minimum of 10 hours of training per week; (2) tested negative for performance-enhancing drugs, and (3) not taking any medications. Subjects were instructed to avoid physical activity in the 48 hours prior to the tests and to refrain from consuming alcohol and caffeine for at least 24 hours. After being informed on the objectives and the research procedures, participants signed the consent document. The study was approved by the Bioethics Committee of the University of Bologna (No. 25027).

### 2.2. Procedures

The participants visited the laboratory three times. All tests were performed at the same time of the day (9:00–12:00 AM), in a quiet room with stable temperature (21 °C; 52% of humidity). On the first visit, subjects performed an incremental cycling test to exhaustion on an electronically braked cycle ergometer (LODE Excalibur, Quinton Instrument, Groningen, the Netherlands) to determine the VO_2max_. The expired gas analysis was performed with the Quark CPET device (Cosmed, Pavona, RM, Italy) while subjects cycled at 30 W for 3 min as a warm-up, followed by an instantaneous increase of 1 W every 2s at a cadence between 70–80 rpm [22] The maximal exercise test lasted until VO_2_ plateau was obtained or at least one of the two additional criteria: (i) a plateau of heart rate despite an increased velocity or (ii) exercise cessation due to substantial fatigue. VO_2_ plateau was defined as an increase in VO_2_ ≤ 50 ml min^−1^ during the last 30s despite increased power [23]. Heart rate (HR) was collected using a Polar RS400 downloadable HR monitor (Polar Electro, Lachine, QC). All data collected and analyzed at visit one was used to individualize the load (at 65 and 95% of VO_2max_) of each participant.

**Figure 1:**
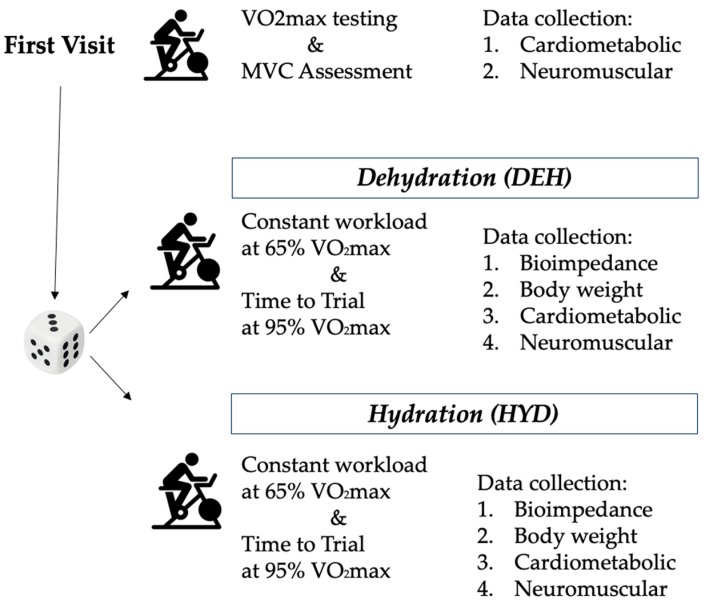
Graphical overview of the testing protocol with the timeline of events.

During the second and third visits, separated by 1 week, the athletes were tested in a randomized, counterbalanced, crossover design (Figure 1); subjects returned to the laboratory and cycled at 65% VO_2max_ for 60 min followed by a TT at 95% VO_2max_, in two different conditions: dehydration (DEH) and hydration (HYD). During the dehydration, subjects completed the trial without ingesting fluids [12,24]. Instead, during the hydration condition, the athletes drank 1 L of water subdivided in 4 steps interspersed by 15 min each other, removing the metabolimeter mask and consuming 0.250 mL of water [25].

At the early morning, before eating breakfast, subjects were weighed (SECA model, Chino, California, USA) 874 with precision to 0.01 kg); then, two hours prior to the cycling, they ingested a meal of 790 kcal; 144 g carbohydrate, 35 g fat, 19 g protein. Additionally, they drank as they normally would the night before and drank 300 mL of water 90 and 45 min before the trial to ensure they were well hydrated before cycling [12]. After the TT, subjects were weighed again to determine their body mass loss over the trial. In addition, to assess fluid loss, bioelectric impedance was measured before cycling (T1) and after 10-minute of shower (T2) according to the procedures reported by Campa et al. [26]. The impedance measurements were performed with a bioimpedance analyzer (BIA 101 Anniversary, Akern, Florence, Italy) using a phase-sensitive device with alternating current at a frequency of 50 kHz. The accuracy of the bioimpedance instrument was validated before each test session following the manufacturer’s instructions. Bioimpedance values were analyzed according to the BIVA method [17]. Bioelectrical phase angle was calculated as the arc tangent of *Xc*/*R* × 180°/π, while the vector length as the hypotenuses of individual impedance values.

Electromyographic (EMG) data were acquired by a Free-EMG (BTS Bioengineering Corp, MA, USA) using Ag/Ag Cl disposable electrodes 32 × 32 mm in a bipolar configuration (RAM Apparecchi Medicali s.r.l., GE, Italy) Electrodes had an active area of 0.8 cm^2^ with an inter-electrode distance of about 2 cm. The skin was shaved and cleaned with ethanol before placing the electrodes to improve the contact with the skin. Electrodes were positioned on the muscular belly of the following muscles of the right leg: vastus medialis (RVM) and biceps femoris (RBF). After placing the electrodes, we acquired the maximum voluntary contraction (MVC) in which each subject had to perform, for 5 s, an isometric contraction against a maximum load using isotonic machines (Technogym, Cesena, FC, Italy). Data was recorded at a sample rate of 1000 Hz and stored for analysis. All raw EMG signals were band pass filtered (20–450 Hz), positively rectified and resampled at 500 Hz. The EMG signals were normalized to the peak of the MVC. Onset and offset times were determined using the double threshold method [27]. The first threshold occurred when the value of the signal exceeded 3SD above the baseline signal, and the second threshold required the signal to remain above this value for at least 30 ms [28]. The criteria used for the first threshold was based on a minimum threshold of 3 SD above the resting baseline signal and a minimum burst duration of 100 ms. The normalized root mean square (RMS) values were calculated in a time window of 100 ms using Matlab (The Mathworks Inc., Natick, Massachusetts, USA). 

In both cycling trials, the energetic cost (EC) was calculated considering data when a metabolic steady state was reached by all subjects [29,30]. The breath-by-breath net oxygen uptake (VO_2NET_, expressed in ml/min/kg), calculated by subtracting the resting VO_2_ (assumed equal to 3.6 ml/min/kg) from VO_2_ values, and the respiratory exchange ratio (RER) were used to determine the instantaneous metabolic power (expressed in W/kg) as VO_2NET_ [(4.94 · RER + 16.04)/60] [30].

### 2.3. Statistical Analysis

Descriptive statistics including means ± SD and data distribution were calculated for all outcome variables. The normal distribution of the data was checked using the Shapiro–Wilk test; thus, the following parametric tests were used: A 2 (condition: hydration/dehydration) × 2 (Time: T1/T2 for bioimpedance and constant workload/Time-to-trial for metabolic and neuromuscular variables) for repeated measure ANOVA was performed. Effect sizes were calculated using partial eta squared (*ηp^2^*). The paired one-sample Hotelling’s T^2^-test was performed to determine if the changes in the mean group vectors (measured between T1 vs. T2) were significantly different from zero (null vector). A 95% confidence ellipse excluding the null vector indicated a significant vector displacement. Data was analyzed with IBM SPSS Statistics version 24.0 (IBM, Chicago, IL, USA), BIVA software [31], and Bodygram TM software (Akern, Florence, Italy). For all tests, statistical significance was set at *p* < 0.05.

## 3. Results

### 3.1. Bioimpedance d-Data

Table 1 shows the changes in the bioelectric values. *R*/*H*, *Xc*/*H* (where *H* represents height measured in meters) and vector length significantly changed (*p* < 0.05) in T2 compared to T1 in the DEH condition. On the contrary, when the athletes performed the trial in the HYD condition, the same values did not change. 

The averages of body weight of athletes in T0 were 74.7 ± 10.4 kg and 74.9 ± 10.3 kg in HYD and DHE conditions, respectively. After both trials body weight showed a significant decrease by measuring 74.5 ± 10.5 kg in HYD (*p* = 0.005) and 73.5 ± 10.3 kg in DEH (*p* < 0.001). When the athletes performed the DEH trial, their body weight decreased by 1.76% ± 0.39%, while when they performed the HYD trial their body weight decreased by 0.3% ± 0.27%.

**Figure 2:**
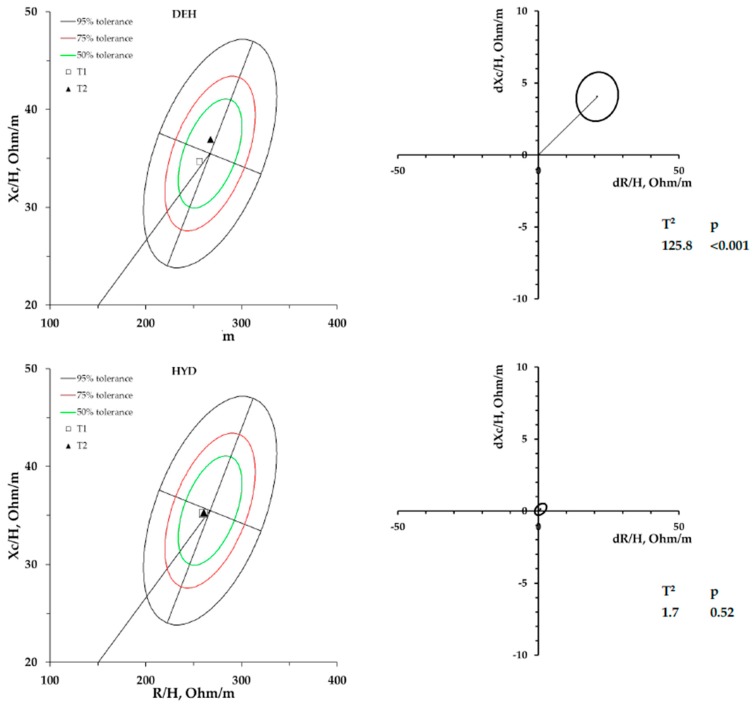
On the left side mean impedance vectors, plotted on the 50%, 75%, and 95% tolerance ellipses of the male athlete’s endurance reference population [32] are displayed both for DEH and HYD trial. On the right side, mean vector displacements and results of the Hotelling’s T^2^ test.

Figure 2 shows the vector displacements (on the left side) and the Hotelling’s T^2^ test results (on the right side) for DEH and HYD conditions, panel A and B, respectively.

### 3.2. Cardiometabolic Data

Table 2 shows the comparisons of exercise duration, power output and cardiometabolic parameters between HYD and DEH on both exercise condition. We found significant differences on time main effect (*p* < 0.001) for all parameters, in which mean values were higher during TT with respect to constant workload at 65% of VO_2max_. Moreover, we found a condition mean effect for duration (*F*_1,8_ = 8.24; *p* = 0.021; *n_p_^2^* = 0.51; 95%CI = 5.3–48.9) and for HR (*F*_1,8_ = 55.21; *p*< 0.001; *n_p_*^2^ = 0.87; 95%CI = 2.9–5.5). Post-hoc analysis showed significant differences between HYD and DEH for duration on TT and for HR during both constant workload and TT (Table 2).

### 3.3. Neuromuscular Data

Table 3 shows the comparison of the normalized RMS values of RVM and RBF muscles between the two-hydration status on both exercise condition. During cycling, significant differences were observed for time and condition main effect on both RVM (Time: *F*_1,7_ = 96.45; *p* < 0.001; *n_p_*^2^ = 0.93; 95%CI = 10.8–17.8; Condition: *F*_1,7_ = 13.16; *p* = 0.008; *n_p_^2^* = 0.65; 95%CI = 2.7–13.2) and RBF muscles (Time: *F_1,7_* = 33.46; *p* = 0.001; *n_p_^2^*= 0.83; 95%CI = 4.0–9.6; Condition: *F*_1,7_= 28.30; *p*= 0.001; *n_p_^2^* = 0.80; 95%CI = 2.8–7.5). RVM and RBF showed higher sEMG activity during the HYD condition on both constant workload at 65% of VO_2max_ and on Time-to-Trial at 95% of VO_2max_ (Table 3).

## 4. Discussion

The aim of the present study was to investigate the effects of dehydration on metabolic and neuromuscular functionality and TT performance during a cycling exercise. This study demonstrated that physiological parameters along with HR and neuromuscular function were altered during a moderate intensity exercise and subsequently TT performance when subjects did not restore fluid loss. Additionally, all metabolic and neuromuscular parameters investigated significantly increased during TT with respect to constant workload at 65% of VO_2max_.

Fluid loss and therefore dehydration during the DEH session was evaluated by BIVA, which identified a significant vector displacement along the major axis of the ellipses; on the contrary, when the athletes restored the fluids lost during the HYD trial, no vector displacement was detected Lengthening of vectors in R-Xc graph represent a decrease in body fluids [16,18]. This evaluation was also supported by the body weight change measured after the DEH trial. Our results showed that although the bioimpedance vectors lengthen as a result of body fluid loss, the slope and phase angle does not change, implying that the ICW/ECW ratio remains unchanged. In this regard, different studies have shown how the phase angle which determines the vector slope of the R-Xc graph, is directly proportionate to the ICW/ECW ratio [33,34,35]. Similar results have been found by Gatterer et al. [16] in regard to dehydration after a running test. No significant difference was observed for metabolic parameters (e.g., VO_2_, RER and EC) between the two trials in both phases of the cycling exercise. However, TT duration and HR variation observed during the cycling performance at 65% and 95%VO_2max_ was significantly different between the two conditions; in fact, a higher HR and a shorter TT performance during DEH session was observed. Our hypothesis was that HR increases during dehydration because of the blood flow redistribution, which causes a high body temperature and a consequent decrease in the stroke and blood volume and consequently compensatory increase in HR. In line with these results, previous studies have documented the resultant tachycardia and diminished stroke volume during dehydration. Our results are similar to those obtained by Logan-Sprenger et al. [12] in an experiment in which 9 subjects who completed two cycling trials lasting 60 minutes at 65% VO_2max_ followed by a TT; as in our study, the subjects showed an increase in HR during dehydration condition compared to when the subjects were hydrated. Furthermore, VO_2_ kinetics, RER, and EC showed tendentially higher values during DEH, although not statistically significant. Probably, a greater percentage of fluid loss is necessary to have significant effects on cardiometabolic parameters during a cycling exercise carried out below the ventilatory threshold (65% VO_2max_).

The RVM and RBF muscle activity was significantly reduced during the two parts of the DEH test, suggesting that fluid loss compromises the muscle power expression during dehydration. The effects of dehydration on muscle performance have been studied using different protocols and measurement techniques. Studies vary in the percentage of lost fluids achieved from 1.7% to 5.8% of body mass reduction [36,37,38,39]. After a literature review, [40] concluded that dehydration consistently attenuates muscle power by approximately 3%. The origin of these reductions has been speculated to reside on alterations in cardiovascular, metabolic or buffering functions. In fact, heat stress, with or without dehydration, compromises blood flow to active muscles and skin during strenuous exercise as the systemic circulation [41].

In a meta-analysis conducted by Goulet [42], it was showed that levels of exercise induced dehydration, similar to those in the present study, did not reduce cycling TT performance. Additionally, in response to a similar hydration status (progressive loss to 2% body weight loss), no significant differences were reported when trained men completed a 40 km cycling TT performance as measured by power output and mean finish time [43]. These records of data suggest the influence of dehydration may, in part, be protocol specific. In our study, athletes cycled for 60 min at 65% VO_2max_ and performed a 95% VO_2max_ TT in two different tests. During the HYD session, in order to restore the fluids lost during exercise, the athletes ingested 0.5 ml of water every 15 min to maintain a body weight similar to that recorded at the baseline. However, to the best of our knowledge, this is the first experiment to use this experimental protocol to ensure a well-hydration status of the athletes during the exercise, and therefore, it is not possible to compare our results with other researches. One of the most common potential limitations is the inherent difficulty in blinding subjects to the fact that they are dehydrating versus rehydrating during a given trial. Another possible limitation in the present study is the participant sample, which may compromise detectable differences among the two hydration conditions in the examined parameters. Nonetheless, other studies evaluating exercise performance and dehydration/rehydration have used samples ranging from n = 6 to 11 [37,44,45].

A strong point of this study is demonstrating how BIVA can be used to monitor the changes in fluids by identifying dehydration. In addition, new evidence regarding the effects of dehydration on physical performance have been provided and these should also be considered in non-athletes because health could be compromised during sports practice.

From the discussion above it is clear that more research is needed to address several remaining questions regarding the potential impact of dehydration on sports performance. Valid and reliable protocols should be developed and used in future studies to ensure that tests are able to detect the effects of fluid loss on central and peripheral parameters. Lastly, future studies should also include female participants to study the impacts of dehydration on both metabolic and neuromuscular variables in subjects of different gender.

## 5. Conclusions

Neuromuscular and metabolic function were altered during a cycling performance when subjects dehydrated versus maintaining a well-hydration status through drinking. The practical application of this study demonstrated that athletes exercising in a dehydrated state significantly decreased physical performances; therefore, attention needs to be paid to strategies to maintain a good-hydration status during exercise. In addition, this study confirms the ability of BIVA to assess body fluid changes even in sports practice.

## Figures and Tables

**Table 1:** Bioimpedance parameters before and after the exercise in both tests.

		T1	T2	ANOVA	
		Mean ± SD	Mean ± SD	Time Effect	Time x Condition
R/H (Ω/m)	HYD	259.9 ± 36.4	260.4 ± 36.9	*F* = 79.1; *p* = <0.001; η^2^_p_= 0.82	*F* = 67.8; *p* = <0.001; η^2^_p_ = 0.79
	DEH	255.6 ± 35.8	267.4 ± 36.1 *		
Xc/H (Ω/m)	HYD	35.2 ± 5.4	35.3 ± 5.4	*F* = 56.3; *p* = <0.001; η^2^_p_= 0.76	*F* = 50.1; *p* = <0.001; η^2^_p_ = 0.73
	DEH	34.7 ± 6.9	36.9 ± 4.9 *		
Vector length (Ω/m)	HYD	262.3 ± 36.7	262.8 ± 37.2	*F* = 82.8; *p* = <0.001; η^2^_p_ = 0.82	*F* = 71.1; *p*= <0.001; η^2^_p_ = 0.79
	DEH	257.9 ± 36.1	269.9 ± 36.4 *		
Phase angle (°)	HYD	7.7 ± 0.5	7.8 ± 0.4	*F* = 3.7; *p* = 0.07; η^2^_p_ = 0.17	*F* = 3.8; *p* = 0.67; η^2^_p_ = 0.17
	DEH	7.7 ± 0.4	7.9 ± 0.4		

Note: HYD: hydration condition; DEH: dehydration condition; * = *p* < 0.05 vs. T1.

**Table 2:** Comparison (mean ± SD) of the exercise duration, power output, and cardiometabolic parameters between hydration status on both exercise conditions.

Variable	Constant Workload (65% VO_2_max)					Time-to-Trial (95% VO_2_max)				
	HYD	DEH	Mean Diff	*p*	d	HYD	DEH	Mean Diff	*p*	d
Duration (min)	-	-	-	-	-	3.19 ± 0.60	2.39 ± 0.61	0.80	* 0.027	1.32
VO_2_ (ml/Kg/min)	31.41 ± 4.28	31.70 ± 4.88	−0.29	0.616	−0.06	43.79 ± 6.51	43.59 ± 8.89	0.21	0.904	0.03
RER	0.93 ± 0.06	0.94 ± 0.04	−0.01	0.400	−0.26	1.10 ± 0.10	1.07 ± 0.07	0.04	0.344	0.43
EC (W/Kg)	9.55 ± 1.40	9.68 ± 1.64	−0.14	0.436	−0.09	14.26 ± 2.41	14.21 ± 3.11	0.05	0.925	0.02
HR (bpm)	136.22 ± 8.75	139.53 ± 8.12	−3.31	* 0.005	−0.39	166.56 ± 8.91	171.65 ± 7.89	−5.09	* 0.002	−0.60
Power (Watt)	147.56 ± 29.64	147.56 ± 29.64	0.00	-	-	280.67 ± 56.24	280.67 ± 56.24	0.00	-	-

Note: HYD: hydration condition; DEH: dehydration condition; RER: respiratory exchange ratio; EC: energetic cost; HR: hear rate; *p*-value and Cohen’s d; *= significant differences between hydration status on each exercise condition (*p* < 0.05).

**Table 3:** Comparison (mean) of the muscle activation between hydration status on both exercise condition.

	Constant Workload (65% VO_2max_)					Time-to-Trial (95% VO_2max_)				
	HYD	DEH	Mean Diff	*p*	d	HYD	DEH	Mean Diff	*p*	d
RVM-RMS (%MVC)	41.96 ± 10.39	34.92 ± 6.53	7.4	0.011 *	0.81	57.30 ± 10.98	48.29 ± 6.13	9.00	0.014 *	1.01
RBF-RMS (% MVC)	20.40 ± 3.88	14.91 ± 2.55	5.49	0.003 *	1.67	26.97 ± 4.41	22.05 ± 4.04	4.92	0.021 *	1.16

Note: HYD: hydration; DEH: dehydration; RVM: right vastus medialis; RBF: right biceps femoris; MVC: maximum voluntary contraction; RMS: root mean square; *p*-value and Cohen’s d; *= significant differences between hydration status on each exercise condition (*p* < 0.05).

## References

[1] Campa F., Semprini G., Júdice P.B., Messina G., Toselli S. (2019). Anthropometry, Physical and Movement Features, and Repeated-sprint Ability in Soccer Players. Int. J. Sports Med..

[2] Piras A., Cortesi M., Campa F., Perazzolo M., Gatta G. (2019). Recovery Time Profiling After Short-, Middle- and Long-Distance Swimming Performance. J. Strength. Cond. Res..

[3] Piras A., Gatta G. (2017). Evaluation of the Effectiveness of Compression Garments on Autonomic Nervous System Recovery After Exercise. J. Strength Cond. Res..

[4] Masento N.A., Golightly M., Field D.T., Butler L.T., van Reekum C.M. (2014). Effects of Hydration Status on Cognitive Performance and Mood. Br. J. Nutr..

[5] Pethick W.A., Murray H.J., McFadyen P., Brodie R., Gaul C.A., Stellingwerff T. (2019). Effects of Hydration Status during Heat Acclimation on Plasma Volume and Performance. Scand. J. Med. Sci. Sports.

[6] Zhang N., Du S.M., Zhang J.F., Ma G.S. (2019). Effects of Dehydration and Rehydration on Cognitive Performance and Mood among Male College Students in Cangzhou, China: A Self-Controlled Trial. Int. J. Environ. Res. Public Health.

[7] Barley O.R., Chapman D.W., Blazevich A.J., Abbiss C.R. (2018). Acute Dehydration Impairs Endurance Without Modulating Neuromuscular Function. Front Physiol..

[8] Castro-Sepulveda M., Cerda-Kohler H., Perez-Luco C., Monsalves M., Andrade D.C., Zbinden-Foncea H., Báez-San Martín E., Ramírez-Campillo R. (2014). Hydration Status after Exercise Affect Resting Metabolic Rate and Heart Rate Variability. Nutr. Hosp..

[9] Georgescu V.P., de Souza Junior T.P., Behrens C., Barros M.P., Bueno C.A., Utter A.C., McAnulty L.S., McAnulty S.R. (2017). Effect of Exercise-Induced Dehydration on Circulatory Markers of Oxidative Damage and Antioxidant Capacity. Appl. Physiol. Nutr. Metab..

[10] Nuccio R.P., Barnes K.A., Carter J.M., Baker L.B. (2017). Fluid Balance in Team Sport Athletes and the Effect of Hypohydration on Cognitive, Technical, and Physical Performance. Sports Med..

[11] McDermott B.P., Anderson S.A., Armstrong L., Casa D.G., Cheuvront S.N., Cooper L., Kenney W.L., O’Connor F.G., Roberts W.O. (2017). National Athletic Trainers’ Association Position Statement: Fluid Replacement for the Physically Active. J. Athl. Train..

[12] Logan-Sprenger H.M., Heigenhauser G.F., Jones G.L., Spriet L.L. (2015). The Effect of Dehydration on Muscle Metabolism and Time Trial Performance during Prolonged Cycling in Males. Physiol. Rep..

[13] Campa F., Silva A.M., Iannuzzi V., Mascherini G., Benedetti L., Toselli S. (2019). The role of somatic maturation on bioimpedance patterns and body composition in male elite youth soccer players. Int. J. Environ. Res. Public Health.

[14] Campa F., Silva A.M., Toselli S. (2018). Changes in Phase Angle and Handgrip Strength Induced by Suspension Training in Older Women. Int. J. Sports Med..

[15] Campa F., Toselli S. (2018). Bioimpedance Vector Analysis of Elite, Subelite, and Low-Level Male Volleyball Players. Int. J. Sports Physiol. Perform..

[16] Gatterer H., Schenk K., Laninschegg L., Lukaski H., Burtscher M. (2014). Bioimpedance Identifies Body Fluid Loss after Exercise in the Heat: A Pilot Study with Body Cooling. PLoS ONE.

[17] Piccoli A., Rossi B., Pillon L., Bucciante G. (1994). A New Method for Monitoring Body Fluid Variation by Bioimpedance Analysis: The RXc Graph. Kidney Int..

[18] Campa F., Matias C.N., Marini E., Heymsfield S.B., Toselli S., Sardinha L.B., Silva A.M. (2019). Identifying Athlete Body-Fluid Changes During a Competitive Season With Bioelectrical Impedance Vector Analysis. Int. J. Sports Physiol. Perform..

[19] Marini E., Campa F., Buffa R., Stagi S., Matias C.N., Toselli S., Sardinha L.B., Silva A.M. (2020). Phase angle and bioelectrical impedance vector analysis in the evaluation of body composition in athletes. Clin. Nutr..

[20] Lukaski H.C., Piccoli A., Preedy V. (2012). Bioelectrical impedance vector analysis for assessment of hydration in physiological states and clinical conditions. Handbook of Anthropometry.

[21] Muth T., Pritchett R., Pritchett K., Depaepe J., Blank R. (2019). Hydration Status and Perception of Fluid Loss in Male and Female University Rugby Union Players. Int. J. Exerc. Sci..

[22] Piras A., Persiani M., Damiani N., Perazzolo M., RaffI M. (2015). Peripheral heart action (PHA) training as a valid substitute to high intensity interval training to improve resting cardiovascular changes and autonomic adaptation. Eur. J. Appl. Physiol..

[23] Astorino T.A., Willey J., Kinnahan J., Larsson S.M., Welch H., Dalleck L.C. (2005). Elucidating determinants of the plateau in oxygen consumption at VO2max. Br. J. Sports Med..

[24] Holland J., Skinner T.L., Irwin C.G., Leveritt M.D., Goulet E.D.B. (2017). The Influence of Drinking Fluid on Endurance Cycling Performance: A Meta-Analysis. Sports Med..

[25] Backes T.P., Fitzgerald K. (2016). Fluid Consumption, Exercise, and Cognitive Performance. Biol. Sport.

[26] Campa F., Gatterer H., Lukaski H., Toselli S. (2019). Stabilizing Bioimpedance-Vector-Analysis Measures With a 10-Minute Cold Shower After Running Exercise to Enable Assessment of Body Hydration. Int. J. Sports Physiol. Perform..

[27] Lee T.Q., Yang B.Y., Sandusky M.D., McMahon P.J. (2001). The effects of tibial rotation on the patellofemoral joint: assessment of the changes in in situ strain in the peripatellar retinaculum and the patellofemoral contact pressures and areas. J. Rehabil. Res. Dev..

[28] Kamen G., Gabriel D.A. (2010). EMG signal processing. Essentials of Electromyography.

[29] Piras A., Campa F., Toselli S., Di Michele R., Raffi M. (2019). Physiological responses to partial-body cryotherapy performed during a concurrent strength and endurance session. Appl. Physiol. Nutr. Metab..

[30] Piras A., Raffi M., Atmatzidis C., Merni F., Di Michele R. (2017). The energy cost of running with the ball in soccer. Int. J. Sports Med..

[31] Piccoli A., Pastori G. (2002). BIVA Software. www.renalgate.it/formule_calcolatori/BIVAguide.pdf.

[32] Campa F., Matias C., Gatterer H., Toselli S., Koury J.C., Andreoli A., Melchiorri G., Sardinha L.B., Silva A.M. (2019). Classic Bioelectrical Impedance Vector Reference Values for Assessing Body Composition in Male and Female Athletes. Int. J. Environ. Res. Public Health.

[33] Gonzalez M.C., Barbosa-Silva T.G., Bielemann R.M., Gallagher D., Heymsfield S.B. (2016). Phase angle and its determinants in healthy subjects: influence of body composition. Am. J Clin. Nutr..

[34] Francisco R., Matias C.N., Santos D.A., Campa F., Minderico C.S., Rocha P., Heymsfield S.B., Lukaski H., Sardinha L.B., Silva A.M. (2020). The Predictive Role of Raw Bioelectrical Impedance Parameters in Water Compartments and Fluid Distribution Assessed by Dilution Techniques in Athletes. Int. J. Environ. Res. Public. Health.

[35] Toselli S., Marini E., Maietta Latessa P., Benedetti L., Campa F. (2020). Maturity Related Differences in Body Composition Assessed by Classic and Specific Bioimpedance Vector Analysis among Male Elite Youth Soccer Players. Int. J. Environ. Res. Public Health.

[36] Bowtell J.L., Avenell G., Hunter S.P., Mileva K.N. (2013). Effect of Hypohydration on Peripheral and Corticospinal Excitability and Voluntary Activation. PLoS ONE.

[37] Minshull C., James L. (2013). The Effects of Hypohydration and Fatigue on Neuromuscular Activation Performance. Appl. Physiol. Nutr. Metab..

[38] Pallares J.G., Martinez-Abellan A., Lopez-Gullon J.M., Morán-Navarro R., De la Cruz-Sánchez E., Mora-Rodríguez R. (2016). Muscle Contraction Velocity, Strength and Power Output Changes Following Different Degrees of Hypohydration in Competitive Olympic Combat Sports. J. Int. Soc. Sports Nutr..

[39] Schoffstall J.E., Branch J.D., Leutholtz B.C., Swain D.E. (2001). Effects of Dehydration and Rehydration on the One-Repetition Maximum Bench Press of Weight-Trained Males. J. Strength Cond. Res..

[40] Judelson D.A., Maresh C.M., Anderson J.M., Adams W.M., Armstrong L.E., Baker L.B., Burke L., Cheuvront S., Chiampas G., González-Alonso J. (2007). Hydration and muscular performance: does fluid balance affect strength, power and high-intensity endurance?. Sports Med..

[41] Crandall C.G., Gonzalez-Alonso J. (2010). Cardiovascular Function. In the Heat-Stressed Human. Acta Physiol..

[42] Goulet E. (2013). Effect of Exercise-Induced Dehydration on Endurance Performance: Evaluating the Impact of Exercise Protocols on Outcomes Using a Meta-Analytic Procedure. Br. J. Sports Med..

[43] Berkulo M.A., Bol S., Levels K., Lamberts R.P., Daanen H.A.M., Noakes T.D. (2016). Ad-Libitum Drinking and Performance during a 40-Km Cycling Time Trial in the Heat. European. J. Sports Sci..

[44] Buono M.J., Wall A.J. (2000). Effect of Hypohydration on Core Temperature during Exercise in Temperate and Hot Environments. Eur. J. Appl. Physiol..

[45] Laitano O., Kalsi K.K., Pearson J., Lotlikar M., Reischak-Oliveira A., González-Alonso J. (2012). Effects of Graded Exercise-Induced Dehydration and Rehydration on Circulatory Markers of Oxidative Stress across the Resting and Exercising Human Leg. European. J. Appl. Physiol..

